# Elexacaftor/Tezacaftor/Ivacaftor in Patients with Cystic Fibrosis Homozygous for the *F508del* Mutation and Advanced Lung Disease: A 48-Week Observational Study

**DOI:** 10.3390/jcm11041021

**Published:** 2022-02-16

**Authors:** Vincenzo Carnovale, Paola Iacotucci, Vito Terlizzi, Carmela Colangelo, Lorenza Ferrillo, Angela Pepe, Michela Francalanci, Giovanni Taccetti, Serena Buonaurio, Assunta Celardo, Laura Salvadori, Giovanni Marsicovetere, Michele D’Andria, Nicola Ferrara, Donatello Salvatore

**Affiliations:** 1 Cystic Fibrosis Centre, Adult Unit, Department of Translational Medical Science, University of Naples “Federico II”, 80131 Naples, Italy; paola.iacotucci@unina.it (P.I.); lorenzaferrillo@gmail.com (L.F.); serenabuonaurio@libero.it (S.B.); susy.celardo@libero.it (A.C.); salvadori.laura@libero.it (L.S.); nicola.ferrara@unina.it (N.F.); 2 Cystic Fibrosis Centre, Department of Paediatric Medicine, Anna Meyer Children’s University Hospital, 50139 Florence, Italy; vito.terlizzi@meyer.it (V.T.); michela.francalanci@meyer.it (M.F.); giovanni.taccetti@meyer.it (G.T.); 3 Cystic Fibrosis Centre, Hospital San Carlo, 85100 Potenza, Italy; c.colangelo@tiscali.it (C.C.); angpepe01@gmail.com (A.P.); marsicogio76@gmail.com (G.M.); micheledandria@gmail.com (M.D.); saverdon@gmail.com (D.S.); 4 Department of Medicine, Surgery and Dentistry “Scuola Medica Salernitana”, Postgraduate School of Pediatrics, University of Salerno, 84081 Baronissi, Italy

**Keywords:** elexacaftor/tezacaftor/ivacaftor, advanced lung disease, cystic fibrosis

## Abstract

Background: Elexacaftor/tezacaftor/ivacaftor (ETI) is the newest cystic fibrosis transmembrane conductance regulator (*CFTR*) modulator drug approved for the treatment of patients with cystic fibrosis (pwCF) aged ≥6 years with at least one copy of the *F508del* mutation (*F*) in the *CFTR* gene or another mutation that is responsive to treatment with ETI. This study determined the effectiveness and safety of ETI in a cohort of severely affected pwCF with an *F/F* genotype. Methods: Retrospective observational study in *F/F* pwCF treated for 48 weeks, enrolled in an ETI managed access program available to subjects with advanced lung disease (ppFEV_1_ < 40). Twenty-six patients from three centres were included. The main outcomes included lung function, sweat chloride concentration (SCC), nutrition, frequency of pulmonary exacerbations (PEx), CFQ-R, and safety. Results: ppFEV_1_ improved by 12.06 (95%CI 8.54, 15.57) from baseline after 4 weeks of treatment with ETI, 15.32 (11.3, 19.34) after 24 weeks, and 14.48 (10.64, 18.32) after 48 weeks. The increase in FEV_1_ was accompanied by a decrease in SCC, improvement of BMI, and noticeable reduction in PEx. An overall good safety profile was observed. Conclusions: In *F/F* pwCF with advanced lung disease with an *F/F* genotype, ETI was safe and associated with clinical improvement.

## 1. Introduction

The combinations of the cystic fibrosis transmembrane conductance regulator (*CFTR*) correctors lumacaftor and tezacaftor with the potentiator ivacaftor were the first *CFTR* modulator drugs approved for the treatment of patients with cystic fibrosis (CF) homozygous for the common *F508del* mutation. In clinical trials, lumacaftor-ivacaftor and tezacaftor-ivacaftor showed a moderate improvement in mean forced expiratory volume in one second (FEV_1_ % predicted), improvement in body mass index (BMI), and a reduction in PEx [1,2,3,4,5,6,7]. To further enhance the modulation of *F508del*-CFTR, it was hypothesised that the addition of a second corrector to a corrector–potentiator combination, acting with a complementary mechanism of action, would be necessary to more fully restore *CFTR* processing and trafficking. Recent randomised clinical trials showed that treatment with a triple combination of *CFTR* modulators, two correctors (elexacaftor-tezacaftor), and the potentiator ivacaftor, resulted in quick and sustained improvements of lung function and nutritional status and reduced the rate of PEx patients carrying at least one *F508del* mutation [8,9,10]. In particular, the RCT involving patients homozygous for the *F508del* mutation showed these encouraging results comparing the triple combination treatment (TCT) with the double treatment tezacaftor-ivacaftor and not with a placebo [8]. These results have led to the approval of elexacaftor-tezacaftor-ivacaftor (ETI) by the US Food and Drug Administration and the European Medicines Agency for patients with eligible *CFTR* genotypes and aged 6 years and older.

Patients with advanced pulmonary disease (e.g., percent predicted FEV_1_ < 40, or under evaluation for lung transplantation) are usually excluded from the Phase III clinical trials, mainly because of comorbidities [11]. No trials have been conducted in pwCF with advanced disease and long-term evaluations are also lacking in this specific subgroup. A managed access program was started in 2019 by Vertex Pharmaceuticals to give the patients homozygous for the *F508del* mutation and with advanced lung disease the opportunity to receive the medication free of charge whilst awaiting national reimbursed commercialisation. The aim of this study was to describe the real-world safety and effectiveness over 48 weeks of ETI treatment in a cohort of *F/F* pwCF.

## 2. Materials and Methods

### 2.1. Study Design and Outcome Measures

Three Italian CF centres participated in this retrospective observational study recruiting 26 subjects, enrolled in an ETI managed access program between October 2019 and July 2020. 

A core set of clinical assessments were conducted at weeks 4, 12, 24, and 48: lung function, SCC, and BMI. The main outcome measure was absolute change from baseline in percentage of predicted FEV_1_ (ppFEV_1_). The secondary outcomes were the absolute change from baseline in *SCC* and in BMI (the weight in kilograms divided by the square of the height in meters) and the number of PEx through the 48 weeks of treatment. The Cystic Fibrosis Questionnaire–Revised (CFQ-R) was administered at weeks 4, 24, and 48, and the absolute change from baseline in the respiratory domain score (range, 0 to 100, with higher scores indicating a higher patient-reported quality of life with regard to respiratory symptoms; minimum clinically important difference, 4 points) as compared. Further end points included safety and side-effect issues.

### 2.2. Study Population

Criteria for entering the managed access program included homozygosity for the *F508del* mutation, together with treatment for at least 6 months with lumacaftor-ivacaftor (LI) or with tezacaftor-ivacaftor, and with severe lung disease (FEV_1_ < 30% predicted, or FEV_1_ < 40% predicted but rapidly declining and/or with ≥6 PEx in the last 12 months) or with the condition of discontinued treatment because of adverse events or drug–drug interactions. Key exclusion criteria included mechanical ventilation, severe liver disease with or without hepatic impairment, history of solid organ or haematological transplantation, history of drug or alcohol abuse in the past year, pregnancy, and sexually active patients of reproductive potential who are not willing to use appropriate contraception methods. Treatment was administered orally according to the manufacturer’s recommendations (200 mg ELX/100 mg TEZ/150 mg IVA in the morning and 150 mg IVA in the evening). Maintenance medications at baseline were continued throughout the study. The COVID-19 pandemic did not affect this study. The scheduled encounters were regularly performed, and no missing data was present.

### 2.3. Methods

Clinical and laboratory data (aspartate aminotransferase, alanine aminotransferase, bilirubin, and creatine-phosphokinase) were collected at baseline and at weeks 4, 12, 24, and 48 following the initiation of ETI treatment. Data of PEx and use of antibiotics were recorded for 48 weeks before initiating ETI. PEx was defined as previously described [12].

To minimise the likelihood that baseline data would be affected by inter-current clinical events, the best-recorded values during the 3 months before initiating the treatment were defined as baseline. 

Vertex Pharmaceuticals played no role in the conceptualisation of this study, in the formal analysis of data, or in the writing and submission of this manuscript.

The study protocol complied with the revised Declaration of Helsinki, and all the recruited subjects provided written informed consent. 

### 2.4. Analysis

Results are presented as means and standard deviations (SDs), means and 95% confidence interval (CI), or medians and range, as appropriate. Data were compared using the Wilcoxon signed rank test, paired *t*-test, or Fisher’s test as appropriate. *p*-values less than 0.05 were considered significant.

## 3. Results

Twenty-six subjects (12 females, 46.2%) were recruited. The median (range) age was 31.1 years (20.8–48.3 years). All patients included in this report had pancreatic insufficiency; CF-related diabetes (CFRD) affected 11 (42.3%) patients. The baseline demographics and characteristics of the study population are described in Table 1. All but one of the patients were on treatment with L/I. One subject had discontinued L/I because of adverse events. No patient was on the lung transplantation waiting list.

### 3.1. Changes Following ETI Treatment

#### 3.1.1. Lung Function

**Figure 1:**
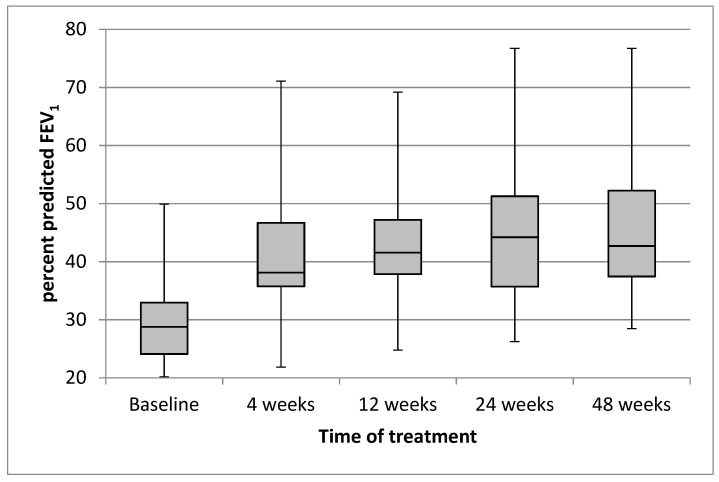
Effect of treatment with elexacaftor/tezacaftor/ivacaftor on percentage of predicted FEV_1_ from baseline through to week 48. Each box and whisker plot shows the position of the minimum, lower quartile, median, upper quartile, and maximum of the data, at baseline and at different times of treatment.

The mean (SD) value of the ppFEV_1_ was 29.9 (8.4) at baseline, and it quickly increased to 41.9 (11.5) after 4 weeks of treatment with ETI. The trend of improvement continued through to week 48 (Figure 1).

The mean (95% CI) absolute improvement in ppFEV_1_ was 12.06 (8.54, 15.57; *p* < 0.0001) after 4 weeks of treatment with ETI, 13.22 (9.47; 16.98; *p* < 0.0001) after 12 weeks, 15.32 (11.3, 19.34; *p* < 0.0001) after 24 weeks, and 14.48 (10.64, 18.32; *p* < 0.0001) after 48 weeks. The changes following the treatment of ppFVC had a similar trend to those of ppFEV_1_ (Table 2).

All subjects showed individual positive responses with respect to ppFEV_1_. Seventeen (65.4%) out of twenty-six subjects showed improvements of ppFEV_1_ greater than 10, and six (23.1%) had increases higher than 20.

#### 3.1.2. BMI

Mean (SD) BMI at baseline was 20.9 (2.16) kg/m^2^. BMI was normal (18.5–24.9 kg/m^2^) in 21 subjects (80.8%), and 5 subjects (19.2%) were underweight (BMI < 18.5 kg/m^2^). Mean (SD) BMI increased to 21.1 (3.1) after 4 weeks of ETI and improved further through to week 48, with a mean (SD) final BMI of 23.0 (2.2).

The mean (95% CI) absolute increase in BMI was 0.66 (0.37, 0.95; *p* < 0.0001) after 4 weeks of treatment with ETI, 1.57 (1.19, 1.94; *p* < 0.0001) after 12 weeks, 2.02 (1.56, 2.48; *p* < 0.0001) after 24 weeks, and 2.08 (1.63, 2.52; *p* < 0.0001) after 48 weeks of treatment (Table 3). After 24 weeks of treatment, all the underweight subjects had improved their BMI to the normal range, and three further patients gained overweight. 

#### 3.1.3. Sweat Chloride

The overall mean (SD) SC concentration was 77.5 (35.3) mmol/L at baseline, and it decreased to 35.9 (20.1) mmol/L after 4 weeks of ETI (*p* < 0.0001) and improved further through to week 48, with a mean (SD) SC concentration decrease to 29.2 (19.5) mmol/L (Table 3). 

#### 3.1.4. Rate of Pulmonary Exacerbations

During the 48 weeks prior to starting ETI, 26 subjects suffered from 105 instances of PEx, of which 42 were treated with intravenous antibiotics (IV). Treatment with ETI for 48 weeks resulted in a 97% lower rate of PEx. Twenty-two subjects (84.6%) remained free of PEx during the period of the study, and only one patient needed IV antibiotics for a single cycle. 

#### 3.1.5. CFQ-R Respiratory Domain

The CFQ-R respiratory domain score improved significantly throughout the study period. The median (range) score was 55.5 (27.8, 88.9) at baseline, which improved significantly to 88.9 (66.7, 100) after 4 weeks (*p* < 0.00001), to 94.4 (61.1, 100) after 24 weeks, and was stable at 94.4 (77.8, 100) until 48 weeks of treatment (*p* < 0.00001). The mean (95% CI) improvements compared to baseline values were 28.4 (19.9 to 36.9), 29.1 (20.1.9 to 38.1), and 32.6 (24.6 to 40.1) points after 4, 24, and 48 weeks of treatment, respectively.

#### 3.1.6. Treatment-Related Adverse Events

No interruption of the treatment was needed, and no patients withdrew from the medication. No ETI-related adverse events (i.e., skin rash, testicular pain, arterial hypertension, headache, anxiety, and depression) were reported. No relevant abnormal results of serum chemistry, haematology, coagulation studies, liver function tests, and urinalysis attributable to ETI treatment were reported. No deaths occurred during the study period.

## 4. Discussion

TCT with ETI significantly improved pulmonary function and CFQ-R respiratory domain over 48 weeks in 26 pwCF homozygous for the *F508del CFTR* mutation and advanced lung disease. Moreover, the TCT resulted in a significant reduction in the annual rate of PEx and in the need for antibiotic therapy. Finally, ETI treatment also resulted in improvements of extra-pulmonary outcomes (BMI and SCC).

The pivotal phase III RCT included *F/F* pwCF, and moderate lung disease showed the clinical efficacy of a 4-week treatment of ETI, resulting in an improved ppFEV_1_ (10.0%) and CFQ-R respiratory domain (17.4 points) and an SCC concentration that was 45.1 mmol/L lower compared with an active comparator (tezacaftor plus ivacaftor alone) [8]. Although the RCT excluded patients with more advanced (ppFEV_1_ < 40%) pulmonary disease at the time of screening, a subgroup of 10 enrolled patients had ppFEV_1_ < 40% on day 1 of the study, but no result is reported about this little subgroup. A further phase IIIB RCT including *F/F* pwCF was conducted for 24 weeks, with a fast improvement of ppFEV_1_ (11.2) after 4 weeks of treatment with ETI that sustained through to 24 weeks [13]. Our patients, all but one treated with L/I before beginning TCT, showed a similar increase in pulmonary function in the first 4 weeks of ETI treatment (mean improvement of ppFEV_1_ 12.1) that was sustained through to 24 and 48 weeks (mean improvements of ppFEV_1_ 15.3 and 14.5, respectively).

Several reports from different countries have shown the early effects of ETI in *F/F* pwCF and severe pulmonary disease. In a single-centre open-label cohort study conducted in Ireland, ppFEV_1_, BMI, and PEx frequency improved among 14 patients (8 were homozygous for *F508del* and were previously on CFTR modulator therapy) with ppFEV_1_ < 40 who were treated with ETI, from a mean ppFEV_1_ of 27 to 36 [14]. A large study reported the experience of all French CF centres, with a cohort of 245 patients, with 100 *F/F*. A subgroup of these *F/F* subjects, previously treated with a *CFTR* modulator, demonstrated a mean improvement in ppFEV_1_ of 11 and 13 after TCT for one and three months, respectively [15]. A short-term retrospective study performed across three large academic CF centres in the United States (US) showed a mean increase in ppFEV_1_ of 7.1 in a group of 32 *F/F* subjects after a mean treatment of 39 days [16]. Another retrospective observational study from the US over one year of ETI administration in a mixed population (*F/MF*, *F/F*, *F/G*, *F/RF*) of 22 adult patients with ppFEV_1_ < 40, showed a mean improvement of ppFEV_1_ 5.5 after 1 month and of 7.6 after 12 months [17].

Finally, a recent prospective, large observational study in 487 pwCF aged ≥ 12 years with ≥1 *F508del* allele from the US showed that ETI treatment resulted in large improvements in lung function, respiratory symptoms, and BMI. In particular, 209 *F/F* subjects were present in the study cohort who were previously treated with L/I or T/I. This *F/F* subgroup had a mean (SD) ppFEV_1_ of 81.1 (22.5) when starting the treatment with ETI, and showed a mean increase in ppFEV_1_ of 7.96 after 1 month, 8.63 after 3 months, and 9.21 after 6 months [18].

Our results agree with all these reports with the follow-up period extended over 48 weeks, much longer than previously reported, confirming that the improvements are as clinically significant as sustained (Table 4).

Another relevant aspect is the distribution of individual positive responses of ppFEV_1_ following the ETI treatment. About 81% of patients had an increase in ppFEV_1_ of ≥ 5 (and 65.4% ≥ 10) and only 19% of patients had an increase in ppFEV_1_ of ≤ 5. This result is comparable to that observed in the French cohort, where an increase in ppFEV_1_ of ≥ 5 was present in 91% of patients and ≥ 10 in 69% [19], and this is better than in the US academic study, where at least 5% improvements of FEV_1_ occurred in 64% of subjects [16].

The objective increase in lung function of our patients treated with ETI was also reflected by the subjective evaluation obtained by the CFQ-R respiratory domain score. The mean increase observed after 4 weeks of treatment was 28.4 points, greater than that in the pivotal RCT (16.0 points) [8]. On the other hand, the mean score at baseline (60.4) of subjects in this study was lower than that in the pivotal trial (70.6 to 72.6), showing more severe pulmonary disease. The improvement of the score was sustained during the 48 weeks of follow-up, showing the stabilisation of the lung disease.

The extra pulmonary outcomes (decrease in SCC and BMI gain) in this study were also relevant. The mean SCC decrease observed after 4 weeks of treatment was −32.6 mmol/L, confirming the recovery of *CFTR* activity. The difference is lower than that observed in the pivotal trial (−43.4), but the mean (SD) baseline SCC in our study was lower (77.5 mmol/L (35.3) vs. 90.0 (12.3) and 91.4 (11.0)). The lower baseline SCC in our patients could be due to the prolonged treatment with L/I. Indeed, it is more similar to the mean (SD) baseline SCC (85.6 (15.2)) of the *F/F* subjects recruited in the US observational study, treated with two-drug modulators before the switch to the TCT [18]. Changes in SCC are considered to provide a direct indicator of systemic CFTR function, and this data has been confirmed in parallel measurements of CFTR function in airway and intestinal epithelia in patients with CF and one or two *F508del* alleles [20].

An important endpoint in this study was the effect of the ETI treatment on healthcare utilisation. The number of PEx dramatically decreased by 97% during the 48 weeks of treatment compared with the year prior to the commencement of ETI. About 85% of the patients were free of PEx after initiating ETI, and only one patient needed a single course of IV antibiotic therapy. These results are comparable to those of the open label extension study of the pivotal trial [10] but need to be cautiously interpreted. Indeed, the study period happened at the same time as limitation of contacts during the COVID-19 lockdown measures were imposed. Real-world data from multiple case records with long follow-up periods are mandatory to confirm this trend. These data, combined with clinical trial results, will help to understand the changing healthcare needs of people with CF [21].

The improved use of body energy is proved by the BMI gain. At week 4, treatment with ETI resulted in a mean increase in BMI of 0.66 kg/m^2^ (95% CI 0.37 to 0.95; *p* < 0.0001), similar to that in the phase III trial. The BMI gain showed gradual increase during the follow up, with a mean increase of 2.08 (95% CI 1.63 to 2.52) after 48 weeks of treatment. At this time, no subject was malnourished (they were 19.2% at the starting point), and 15% of the patients were overweight. A similar increase in BMI after using CFTR modulators in severely affected pwCF was described in patients with the F/MF genotype [22] and in patients with mutations determining *CFTR* residual function [23]. Although over the last decades an emphasis has been on promoting BMI gain to optimise pulmonary outcomes, the risk of overweightness/obesity in an adult population should be monitored in the long-term [24,25,26].

About the safety of ETI treatment, over the 48 weeks of treatment, no interruption of the treatment was needed, and no patients withdrew from the medication. The overall safety data of this study was consistent with clinical trials and real-world findings. In our small sample of pwCF over a 48-week period, ETI treatment was not associated with new safety concerns. In any case, the collection of safety data over longer periods of treatment is essential to strengthen these observations.

Real-world evidence data about the effectiveness and safety of new drugs in pwCF with advanced lung disease, who are at high risk of comorbidities and of rapid worsening in pulmonary function and quality of life, are very important because these subgroups of patients are usually not eligible for clinical trials [11,27]. 

This study had the limitations of a retrospective design, including a small number of patients and the lack of a control group. We strove to work out these problems by performing within-group comparisons. Indeed, this was a descriptive, rather than analytical, study. Moreover, quantitative microbiological data and imaging are lacking in this study. There is evidence that pwCF with CFTR gating mutations were less likely to eradicate bacteria colonising their airways, even when treated with the *CFTR* potentiator ivacaftor [28,29]. L/I decreased the total bacterial load and increased the diversity of the airway microbiome in *F508del* homozygous patients [30]. ETI treatment has showed to confer additional benefits relative to previous CFTR modulators in patients with *F508del* homozygosity [8], *F508del*-gating or *F508del*-residual function genotypes [31]. To evaluate the microbiological effect of more effective CFTR rescue, longer observation times are needed.

Longer follow-up periods are required to validate the observed improvements with respect to clinical stability, slowing down or stopping the decline in pulmonary function, reducing the incidence of complications, and improving the survival [32].

## 5. Conclusions

Treatment with ETI was shown to be effective and safe in homozygous *F/F* pwCF with advanced lung disease. Further studies are needed to define whether the slowing down or reversion of severe pulmonary disease could be obtained in pwCF, and whether more people with severe CF will gain life-changing benefits from CFTR modulation.

## Figures and Tables

**Table 1:** Baseline demographics.

Demographics	
Patients	26
Age, years (median, range)	31.1 (20.8–48.3)
Sex, female (*n*, %)	12 (46.2)
FEV_1_ (% predicted, mean, SD)	29.9 (8.4)
FVC (% predicted, mean, SD)	50.9 (11.6)
FEF_25–75_ (% predicted, mean, SD)	12.2 (6.7)
Sweat chloride (mmol/L, mean, SD)	77.5 (35.3)
BMI (kg/m^2^, mean, SD)	20.9 (2.2)
Microbiology	
*Staphylococcus aureus* (%)	9 (34.6)
*Pseudomonas aeruginosa* (%)	19 (73.1)
*Burkholderia cepacia* complex (%)	2 (7.7)
Pancreatic insufficiency (*n*, %)	26 (100)
CFRD (*n*, %)	11 (42.3)
Concomitant medications (*n*, %)	
Bronchodilators	26 (100.0)
Dornase alpha	18 (70.6)
Hypertonic saline	15 (58.8)
Inhaled antibiotics	23 (88.2)
Oxygen	5 (17.6)
Azithromycin	13 (50.0)

Abbreviations: FVC, forced vital capacity; FEV_1_, forced expiratory volume in the first second; FEF_25–75_, forced expiratory flow from 25% to 75% of vital capacity; BMI, body mass index; CFRD, cystic fibrosis-related diabetes; SD, standard deviation.

**Table 2:** Absolute change of lung function after treatment with ETI over the 48-week period.

Variable	After 4 Weeks	After 12 Weeks	After 24 Weeks	After 48 Weeks
FEV_1_ (% Predicted)	12.06 (8.54, 15.57) ^‡^	13.22 (9.47, 16.98) ^‡^	15.32 (11.3, 19.34) ^‡^	14.48 (10.64, 18.32) ^‡^
FVC (% Predicted)	13.08 (8.54, 17.62) ^‡^	14.59 (9.69, 19.49) ^‡^	18.89(14.20, 23.59) ^‡^	18.50(13.64, 23.35) ^‡^

Captions: Data are shown as mean (95% Confidence Interval). Abbreviations: ETI, elexacaftor-tezacaftor-ivacaftor; FEV_1_, forced expiratory volume in the first second; FVC, forced vital capacity. ^‡^ *p* < 0.0001.

**Table 3:** Absolute changes in sweat chloride and BMI over the 48-week treatment period.

Variable	Baseline	After 4 Weeks	After 12 Weeks	After 24 Weeks	After 48 Weeks
Sweat chloride (mmol/L) ^a^	77.5 (35.3)	35.9 (20.1) ^‡^	35.4(16.8) ^‡^	29.0 (11.0) ^‡^	29.2 (19.5) ^‡^
BMI (kg/m^2^) ^a^	20.9 (2.2)	21.1 (3.1) ^‡^	22.4 (2.2) ^‡^	23.1 (2.3) ^‡^	23.0 (2.2) ^‡^

Captions: ^a^ Data are shown as mean (standard deviation). Abbreviations: BMI, body mass index. ^‡^ *p* < 0.0001.

**Table 4:** Comparison of the main results of this study with previous reports.

Reference	Year	Number of Patients	Follow Up Period	ppFEV1 (%)	SCC (mmol/L)	Weight (Kg) and BMI (kg/m^2^)	PEx Requiring Hospitalisations
Carnovale V et al.		26	48 weeks	Absolute change in ppFEV1: 14.48% (10.64 to 18.32)	77.5 ± 35.3 vs. 29.2 ± 19.5	BMI: 20.9 ± 2.2 vs. 23.0 ± 2.2	97% lower rate of PEx
Heijerman, H. et al. [8]	2019	107 ETI group: 55	4 weeks	Absolute change in ppFEV1: 10.4% (8.6 to 12.2)	Absolute change: −43.4 mmol/L (−46.9 to 40.0)	Mean increase in BMI: 0.60 Kg/m^2^	No data
O’Shea KM et al. [14]	2020	14 (57% *F508del* homozygous)	ppFEV1: 26.4 ± 4.2 days SSC: 64 ± 84 days BMI: 62 ± 35 days PEX:4.9 ± 1.94 months	27.3 ± 7.3% basal vs. 36.3 ± 16.5% after treatment	104.9 ± 15.04 vs. 53.6 ± 23.3 (only 11 patients)	BMI: 20.7 ± 3.6 vs. 22.1 ± 3.4 kg/m^2^	0.28 ± 0.17 PEx per month in 12 months prior vs. 0.04 ± 0.07 PEx per month during follow-up period
Burgel PR et al. [15]	2021	236 (40.8% *F508del* homozygous)	3 months	Mean change in ppFEV1: 15.1%	No data	Mean increase in weight: 4.2 Kg	No data
Bermingham B et al. [16]	2021	50 (64% *F508del* homozygous)	ppFEV1: 39.1 ± 24.8 days	Mean change in ppFEV1: 7.9%	No data	No data	No data
Djavid AR et al. [17]	2021	22 (9% F508del homozygous))	12–15 months	Mean change in ppFEV1 in 16/22 patients: 7.6%	No data	Mean increase in BMI: 2.0 Kg/m^2^	Decrease of 2.38 PEx per patient
Nichols DP et al. [18]	2021	487 (48.5% F508del homozygous)	6 months	Mean change in ppFEV1: 9.76	88.0 ± 18.4 vs. 45.7 ± 21.2	BMI: 23.1 ± 4.0 vs. 24.5 ± 4.6	No data
Sutharsan S et al. [13]	2021	175 ETI group: 87	24 weeks	Absolute change in ppFEV1: 11.2% (9.8 to 12.6)	Absolute change: −46.2 mmol/L (−48.7 to 43.7)	Absolute change in BMI: 1.59 Kg/m^2^	No data

Abbreviations: ppFEV_1_, percentage of predicted forced expiratory volume in one second; SCC, sweat chloride concentration; BMI, body mass index; PEx, pulmonary exacerbations; ETI, elexacaftor-tezacaftor-ivacaftor.
